# Long-term monitoring and analysis of Brood X cicada activity by distributed fiber optic sensing technology

**DOI:** 10.1093/jisesa/iead090

**Published:** 2023-11-30

**Authors:** Sarper Ozharar, Jessica L Ware, Yue Tian, Yangmin Ding

**Affiliations:** NEC Laboratories America Inc., 4 Independence Way, Princeton, NJ 08540, USA; American Museum of Natural History, Division of Invertebrate Zoology, New York, NY 10024, USA; NEC Laboratories America Inc., 4 Independence Way, Princeton, NJ 08540, USA; NEC Laboratories America Inc., 4 Independence Way, Princeton, NJ 08540, USA

**Keywords:** Cicada, acoustics, Brood X, fiber optic networks

## Abstract

Brood X is the largest of the 15 broods of periodical cicadas, and individuals from this brood emerged across the Eastern United States in spring 2021. Using distributed acoustic sensing (DAS) technology, the activity of Brood X cicadas was monitored in their natural environment in Princeton, NJ. Critical information regarding their acoustic signatures and activity level is collected and analyzed using standard outdoor-grade telecommunication fiber cables. We believe these results have the potential to be a quantitative baseline for regional Brood X activity and pave the way for more detailed monitoring of insect populations to combat global insect decline. We also show that it is possible to transform readily available fiber optic networks into environmental sensors with no additional installation costs. To our knowledge, this is the first reported use case of a distributed fiber optic sensing system for entomological sciences and environmental studies.

## Introduction

There are 7 species of periodical cicadas, which are endemic to the United States, in the genus *Magicicada* (Davis 1925). These occur in 15 broods distributed from the Mississippi River—Drainage to the East Coast of the United States. Detailed studies regarding the ecology, evolution, and distribution of these taxa are reviewed in Simon et al. (2022), but few studies have yet to collect vast remote distributional data, especially in rural areas. Nymphs develop underground, where they feed on fluid in the xylem of the roots of trees. Nymphs emerge from the ground as adults in each brood, either every 13 or 17 yr, with some stragglers emerging early or late each year (Marshall 2001). Brood X is the largest of the broods, which comprised 3 species: *Magicicada septendecim* (Linnaeus 1758), *Magicicada septendecula*Alexander and Moore, 1962, and *Magicicada cassinii* (Fisher 1852).

In a time of unprecedented insect decline (e.g., Kehoe et al. 2021, Wagner et al. 2021) and changing habitat (Keil et al. 2015, Segan et al. 2016), monitoring insects has been highlighted as a key goal to establish a baseline data for species experiencing the effects of climate change. Monitoring efforts have attempted to track cicada broods systematically since the late 19th century, although indigenous knowledge described brood emergences even before the United States was colonized (Marlatt 1907). More recently, citizen scientists have become involved in *Magicicada* monitoring efforts using the mobile phone applications Cicada Safari and iNaturalist (Kristsky 2021). While such observational data can provide valuable insight into where and when cicadas are emerging, it may be strongly shaped and biased by human activity patterns. It has been more difficult to observe brood behavior continuously, although radar (Kritsky 2021), and sound recordings (Blount 2021) can determine where the cicadas are concentrated. There is a need for a new monitoring method that would allow, in addition to the collection of distribution data, the recording of cicada activity and the intensity of such activity over large areas for long-time durations.

Distributed fiber optic sensing (DFOS) is a new technology that transforms fiber optic cables into sensing elements (Hartog 2017). Historically, it can be traced back to the optical time domain reflectometry (OTDR), which was and still is an important tool for maintaining and monitoring fiber routes. DFOS is a very broad technology that covers multiple different approaches based on what is measured, how it is measured, and what kind of fiber is used. However, 4 major configurations are prevalent; these are distributed temperature sensing to measure the temperature of the fiber (Shiota et al. 1992), distributed vibration sensing to measure vibrations (Juskaitis et al. 1994), distributed strain sensing to measure strain on the cable (Kee et al. 2000), and distributed acoustic sensing (DAS) to measure the acoustic disturbances along a fiber cable route (Pan et al. 2011). Each of these methods has many applications in smart cities, including traffic monitoring, infrastructure monitoring, and even Earthquake detection (Huang et al. 2020, Wellbrock et al. 2019, Ozharar et al. 2021, Nishimura et al. 2021, Ajo-Franklin et al. 2019). There are several advantages of fiber optic sensing compared with traditional sensing methods. One major advantage is that the fiber optic cable, used as the sensing element, is completely passive, and does not require any external power. Another major advantage is that it can easily cover and monitor a long route (~50 km), with high spatial resolution (~1 m). This is identical to installing ~50,000 sensors in the monitored region that are inherently synchronized and do not require onsite power supply.

In this work, a DAS system was used to interrogate standard telecommunication grade outdoor fiber cables installed in our testbed to monitor Brood X *Magicicada* cicadas in situ along a suburban stretch of Central New Jersey over a 16-day period during their 2021 adult cycle. The author’s sampling near this location in both Princeton Junction and Princeton recovered all 3 species, so it is likely that all 3 species was recorded in our study area. Using the DAS data collected from the field, the spectral patterns of cicada sounds were evaluated and a direct relationship between these calls and ambient temperature was observed. We propose the use of DAS as a potential tool for remotely monitoring insects in situ wherever fiber cables are available.

## Distributed Acoustic Sensing Fundamentals

When a highly coherent optical pulse travels along a fiber cable, a small fraction of it backscatters toward the source due to the inherent nonuniformity centers randomly distributed along the fiber cable (Rayleigh 1899). By monitoring the time of arrival of this backscattered light (relative to the pulse launching time), one can calculate at which point along the fiber this backscattered light has originated, since the speed of light inside the fiber cable is known and constant. This principle is also exploited in OTDR devices used to measure the attenuation profile of fiber cables (Barnoski et al. 1976).

Moreover, if there is a strong enough external perturbation, such as an acoustic event, at or near any point along the fiber cable, the generated acoustic pressure wave will cause a time-varying small strain on that point of the fiber cable. These changes in the strain will modulate the local refractive index of the fiber at that point, which in turn will modulate the optical phase of the light backscattered from that point (Bertholds et al. 1988). Since the wavelength of the light is on the order of microns, even minuscule changes in the refractive index result in measurable changes in the optical phase making the system very sensitive. In simple terms, a DAS system measures the optical phase of the backscattered light along the whole fiber length, and keeps track of its change and its origin, hence detecting and locating the acoustic events along the fiber route. The reader should note that, the DAS system can also detect vibration events such as a bird landing on a fiber optic cable, or an animal walking along the fiber optic cable as well. However, since those events are at a single location and for a limited time, such events will not interfere with our study.

**Fig. 1.**
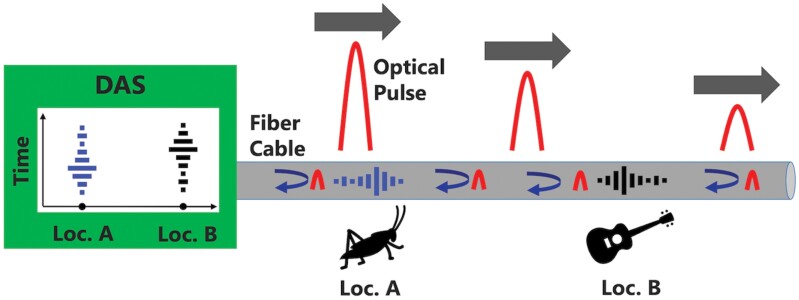
Operation principle of a DAS system. As the optical pulse travels along a fiber cable, it backscatters toward the source. If there are some acoustic sources near the fiber cable, their vibrations modulate the optical phase of this backscattered light; hence, those acoustic signals can be detected and localized.

A simple operation principle of the DAS system is demonstrated in Fig. 1. Multiple acoustic sources (an insect and a guitar in this example) generate time-varying pressure waves at different locations along the fiber, which result in local strain changes at different points on the said fiber. While a single optical pulse travels along and interrogates the fiber, a fraction of that optical pulse backscatters toward the DAS, where the time of arrival and the optical phase of the backscattered light is measured and recorded. The time of arrival carries the information regarding the location, and the optical phase carries information regarding the local strain and therefore acoustic pressure wave. If one interrogates the fiber at a high enough optical pulse repetition frequency, the acoustic pressure wave can be sampled and reconstructed. The DAS can also distinguish multiple acoustic sources along the fiber, provided that they are spaced more than the spatial resolution of the system. Hence, a DAS system transforms a fiber cable into segments of individual acoustic sensors. The length of each segment, in other words, the spatial resolution of the system, depends on the parameters such as the optical pulse width and the chosen gauge length. Also, the optical pulse repetition rate determines the acoustic sampling frequency of the system, since it defines how many times in one second the system interrogates the fiber cable and samples the phase (i.e., acoustic) data.

## Testbed and Data Collection for In Situ Monitoring of Brood X Cicadas

**Fig. 2.**
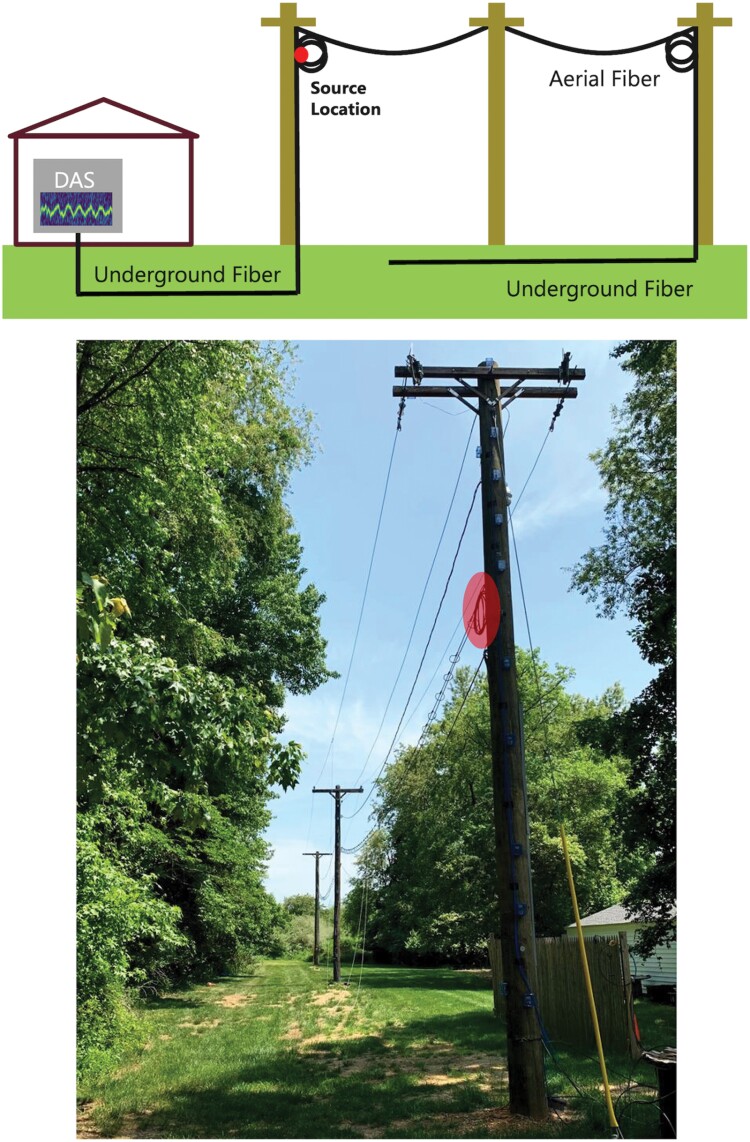
The schematics and a picture of the testbed, the red circle shows the chosen monitoring location in the diagram and the picture.

To monitor Brood X cicadas in their natural environment, we used a standard 36-strand single-mode outdoor-grade figure-8 telecom fiber cable with a 0.25-inch messenger installed in an outdoor testbed. The testbed consists of 3 Class-II-type 35-feet tall utility poles in a linear arrangement and 90 feet apart from each other. The telecom fiber is connected to a DAS system located inside the lab; the first 340 m of the fiber cable is underground, then it rises along the first pole and becomes aerial, and at the third pole it goes underground again. The overall schematics of the testbed with the fiber route and a picture are shown in Fig. 2.

The DAS system used to monitor the fiber route is a commercially available NEC LS3200 model distributed acoustic sensor. During the data collection, the optical pulse width was chosen as 8 ns, and the spatial resolution was set at 1.2234 m. The optical pulse repetition rate was chosen as 20 kHz for short-time duration acoustic recordings, and 2 kHz for long-time duration data recordings to manage the data size. The DAS data were collected from 9 June to 24 June 2021 intermittently. Using the DAS system, the acoustic signal of the cicada group (a deme, or subpopulation of the larger total NJ emergence; we use the term group here for simplicity) was recorded and analyzed in situ.

## Acoustic Analysis of Brood X Cicadas

**Fig. 3.**
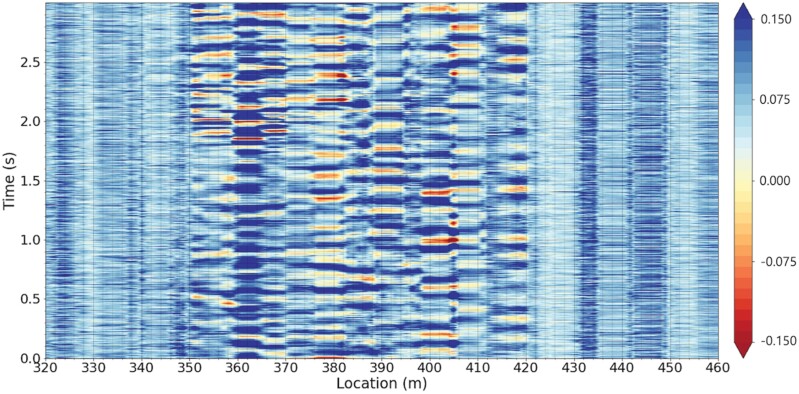
An exemplary 3-second-long spatiotemporal plot collected from the testbed.

As discussed above, the DAS system can detect the acoustic signals along the whole fiber route provided that they are loud enough or close enough to cause minuscule modulations on the optical fiber. Figure 3 shows an exemplary 3-s-long spatiotemporal trace of the data collected from the fiber route at 20 kHz sampling rate. In this spatiotemporal figure, the x-axis shows the position along the fiber in meters, the y-axis shows the time, and the signal amplitude is color coded. By examining the figure visually, one can tell that the aerial section of the fiber (345- to 420-m range) is louder than the underground sections.

For the acoustic analysis of the cicada sounds, a single point along the fiber was selected based on the detected signal quality and power. The selected location is at the 345-m point along the fiber, which corresponds to a fiber coil at the first utility pole; hence, it is an aerial point and collects the cicada data accordingly. As expected, fiber coils are more sensitive and are preferred to detect external vibrations. This fiber coil was highlighted by red circles in Fig. 2 as well.

## Short-Term High-Frequency Monitoring Results

**Fig. 4.**
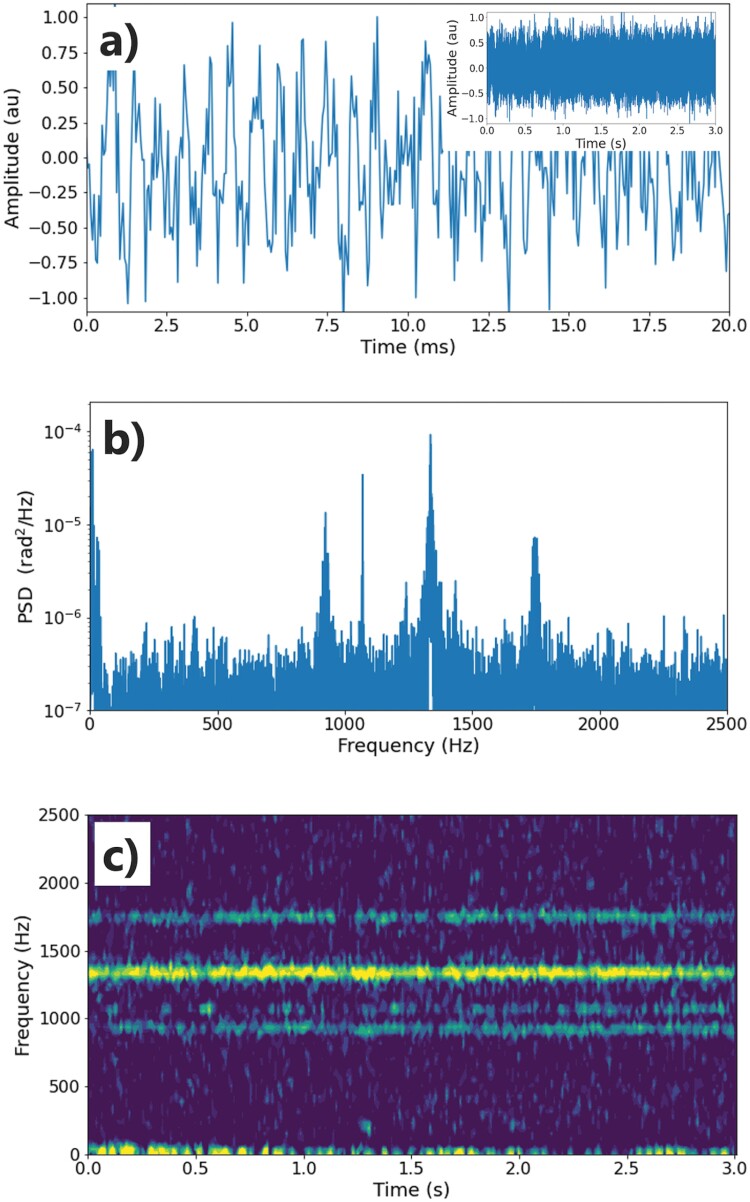
Time domain (a), power spectral density (b), and the short-time Fourier transform (c) of a 3-s-long data recorded by the aerial section of the fiber cable through the DAS.

Cicada signals were recorded successfully over the duration of the experiment. Figure 4a shows the 20 ms long and also the 3-s-long (inset) time domain acoustic signals recorded by the aerial fiber. Figure 4b shows the power spectral density of this 3-s-long signal.

Lastly, Fig. 4c shows the short-time Fourier transform of the same signal. The peak frequency component of the cicada group’s sound captured by the aerial fiber cable was at 1.33 kHz. To confirm the frequency signature of the cicada chorusing captured by the fiber sensing system, a direct in situ audio measurement of the same chorusing was calculated and the peak frequencies were shown to be matched.

Due to the large coverage of the fiber, the system can record multiple locations simultaneously as discussed earlier. However, depending on the cable installation and location, each point of the fiber may not have the same sensitivity to external disturbances; hence, fiber coils are more sensitive than linear cables. The aerial part of the fiber detected and recorded the collective sound of the nearby cicada group, i.e., the cacophony of the cicada deme/population, instead of the sound of an individual cicada. In contrast, the underground cable is acoustically insulated, and the cacophony sound of the cicada population cannot reach the underground fiber to generate a detectable signal.

## Long-Term Low-Frequency Monitoring Results

**Fig. 5.**
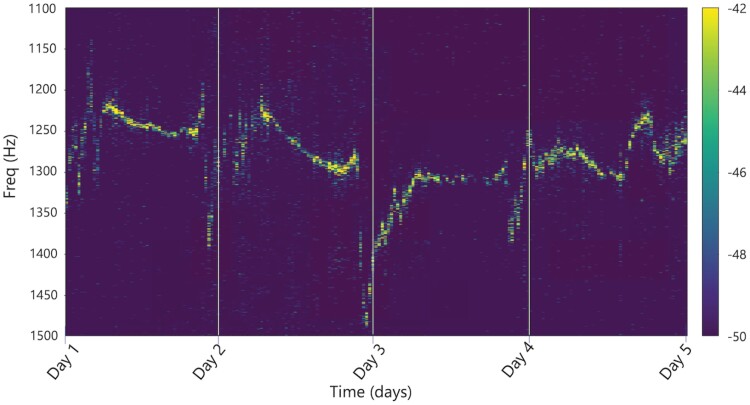
The cicada group spectrum from 9 June until 13 June.

As noted above, the DAS system monitored the testbed for a temporal duration of 16 days, intermittently from 9 June to 24 June 2021. For this long-term data collection, we used a sampling frequency of 2 kHz to reduce the data size. Even though the corresponding Nyquist frequency is lower than the cicada peak frequencies and causes aliasing, the energy of the cicada group is still present in the recorded signal and its frequencies can simply be recovered by the aliasing frequency formula. Figure 5 illustrates how the peak spectral frequency of the cicada group has varied over the 5-day period from 9 June to 13 June captured by the aerial fiber after frequency correction.

**Fig. 6.**
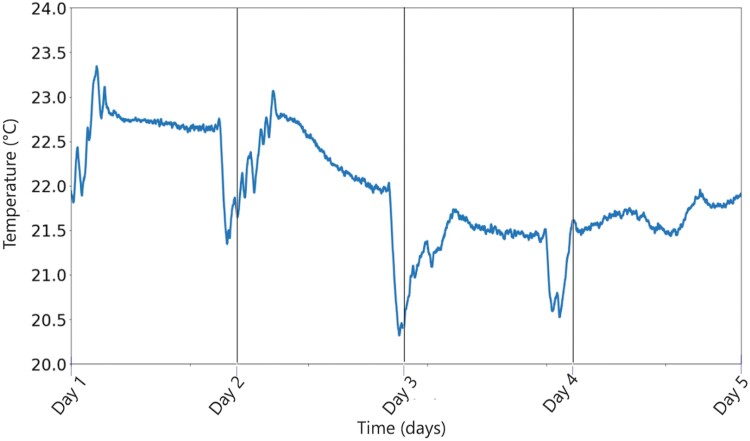
The ambient temperature of the testbed over the time span from 9 June to 13 June.

The day markers correspond to the noontime of each day, and as seen in this figure, the peak frequency of the cicada group varies from a maximum of 1,500 Hz to a minimum of 1,200 Hz in that time frame. Figure 6, on the other hand, shows how the temperature of the testbed was varied at the same location for the same time period, measured by a weather station installed at Pole 1 in our testbed.

When comparing Figs. 5 and 6, it is clear to observe the definite relation between the ambient temperature and the peak frequency of the cicada group. To our knowledge, this is the first long-term result showing such a relationship. The reader should note that a very detailed previous study in the literature shows that there is no relation between air temperature and the cicada chorus pitch, which may seem like a contradiction at first sight (Marshall and Cooley 2000). However, it is to be noted that the relation presented in this paper is not a one-to-one function between ambient temperature and the peak frequency, but rather a trend indicator showing how the peak frequency of the group moves with varying ambient temperature. In other words, a given temperature does not correspond to a unique peak frequency of the group, but as the temperature rises (drops), the peak frequency drops (rises) as well, but the amount of change is not related to the temperature and may need further study. For example, the average temperature from Day 1 to Day 2 is higher than the average temperature from Day 4 to Day 5; however, both days have similar frequencies. The reader should also note that these observations are done in a limited temperature range between 20 and 23.5 °C. Hence, we claim a trend between temperature change and frequency, and not a correlation between temperature and frequency.

**Fig. 7.**
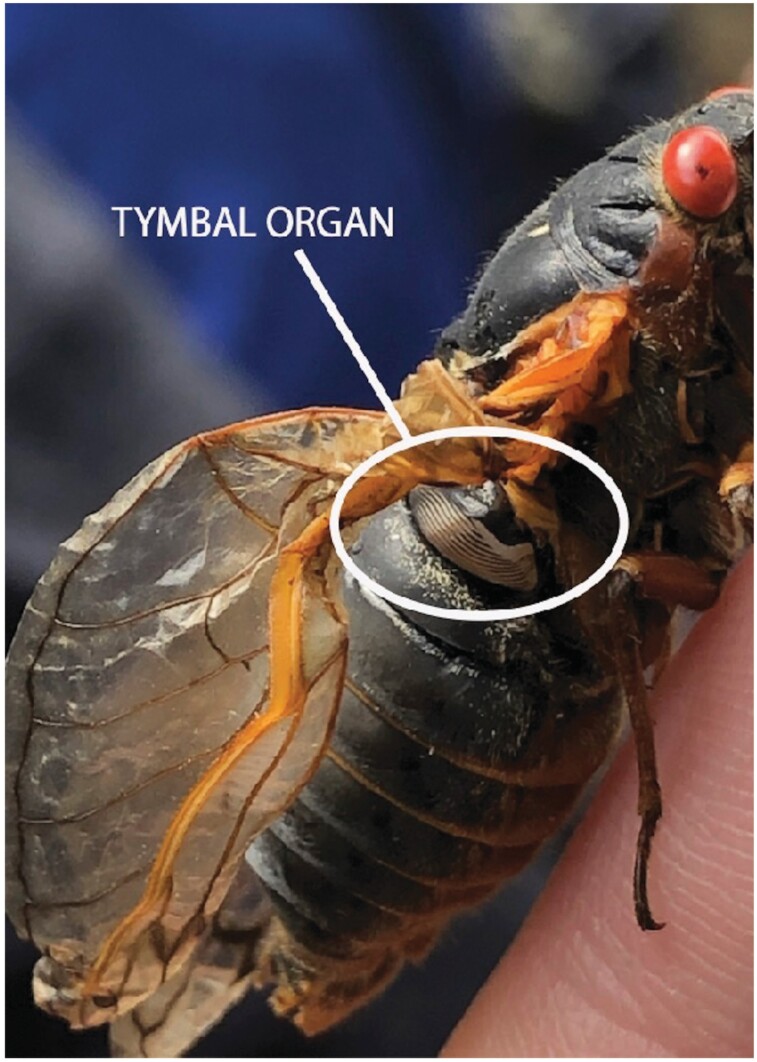
A male Magicicada from Brood X, photographed in Princeton, NJ. The white circle indicates the location of the tymbal organ. Note: male has deformation of its wings.

Male cicadas produce sound using a tymbal organ, as shown in Fig. 7, which is a membrane that vibrates to produce sound (Young and Kritsky 1987, Young et al. 1995). In general, males chorus more on days that have little rain and wind, with their peak chorus volume usually in the mid-afternoon (Alexander et al. 1958). The sound frequency, at which a cicada calls, is influenced by body size and the tymbal organ vibration period (Pringle et al. 1954, Daniel et al. 1993, Bennet-Clark et al. 1994); as ambient temperature increases to 30 °C chorusing volume usually increases, up to 35 °C when shade-seeking behavior is common (Heath et al. 1967).

## Cicada Activity Decay Over Time

**Fig. 8.**
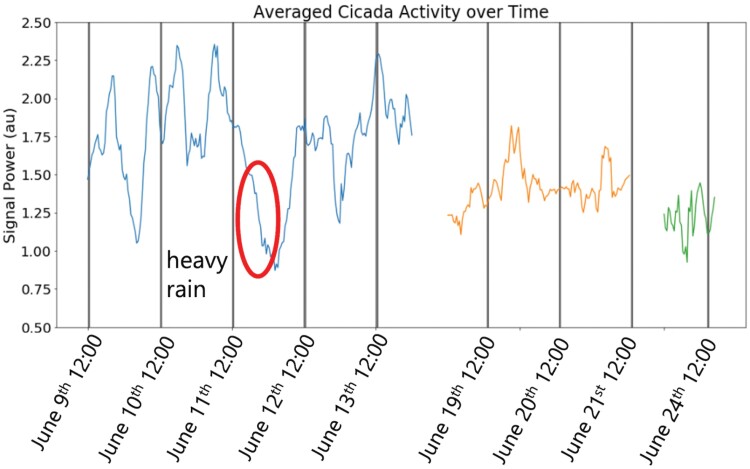
The total integrated signal power of the testbed over 3 temporal spans.

Using the DAS system as a long-term monitoring tool, not just the frequency but also the total sound energy of the cicada population was calculated as a function of time as shown in Fig. 8. This figure shows the integrated total energy in the frequency range of 1,100–1,500 Hz, covering 3 temporal spans. The first span is from 9 to 13 June, the second span is from 19 to 21 June, and the last span covers several hours on 24 June. Since the DAS device is not calibrated for acoustic signal power and the monitored frequency range is not unique to cicadas only, this is a relative measurement and shows the overall decay in cicada activity over time, or to be more precise the overall decay in the detected acoustic power in the frequency band of 1,100–1,500 Hz. To be noted, the heavy rain on the afternoon of 11 June resulted in a dip in the cicada activity which was recovered the next day.

Indeed, the active calling period for the periodical cicadas is relatively short, compared to their 17-yr juvenile stage, lasting typically 2–3 wk in duration. Cicadas emerge after the juvenile period underground and usually take between 4 and 5 days to begin chorusing depending on the ambient temperature. The chorusing period ranges from approximately 1 month. Here, the waning sound signal power in their chorusing toward the end of the reproductive period was recorded. The females lay eggs by making slits in the branches of trees using an egg-laying apparatus called an ovipositor. After a period of egg laying, the females die. The eggs hatch 6-8 wk later, and nymphs drop to the soil to begin their 17-yr juvenile period (Williams and Simon 1995). We hope that these observational data will allow for comprehensive ethology on this charismatic insect over a long time and a large area.

Our results show the feasibility of in situ monitoring cicadas via DFOS technology. We want to stress that all the results presented in the paper are obtained by the use of standard outdoor telecommunication fibers, and not some specialty fibers. Moreover, even the fiber cables in our testbed are installed in the same way as in actual field fibers. Hence, our proposed method purely relies on readily existing fiber cable networks and does not require any special fibers nor a specific way of fiber installation in the field. We hope that it may be beneficial for long-term monitoring of cicadas in large areas and initiate a baseline for a more quantitative analysis of the insect decline. This novel use case also allows one to map cicada activity in real-time along an interrogated fiber route. Future studies should compare cicada activity using these fibers across their geographic range and should test whether the activity of single individuals can be recorded over time via underground fibers.
